# Efficacy of a synthetic peptide *Chlamydia pecorum* major outer membrane protein vaccine in a wild koala (*Phascolarctos cinereus*) population

**DOI:** 10.1038/s41598-023-42296-7

**Published:** 2023-09-12

**Authors:** Sarah J. Simpson, Damien P. Higgins, Peter Timms, Valentina S. A. Mella, Mathew S. Crowther, Cristina M. Fernandez, Clare McArthur, Samuel Phillips, Mark B. Krockenberger

**Affiliations:** 1 Sydney School of Veterinary Science, The University of Sydney, Sydney, NSW 2006 Australia; 2 Centre for Bioinnovation, University of the Sunshine Coast, Sippy Downs, QLD 4556 Australia; 3 School of Life and Environmental Sciences, The University of Sydney, Sydney, NSW 2006 Australia

**Keywords:** Infectious diseases, Vaccines

## Abstract

Chlamydiosis is a significant disease affecting Eastern Australian koala (*Phascolarctos cinereus*) populations, impacting individual animal welfare and fecundity and therefore influencing population dynamics. The aim of this study was to investigate the effect of a synthetic peptide vaccine based on 4 components of the *Chlamydia pecorum* major outer membrane protein (MOMP), over an 18-month period in a koala population severely impacted by chlamydiosis. Wild koalas were recruited into a vaccination or a placebo treatment group on a random allocation, then followed through a period of 18 months, with recapture at 6 monthly intervals. Vaccination did not alter clinical disease expression or chlamydial shedding from the ocular or urogenital sites. Vaccination did not stimulate a significant plasma anti-MOMP IgG response, when compared to the placebo group. There was no significant effect of vaccination on IFN-γ and IL-17A mRNA expression of peripheral blood lymphocytes when stimulated with rMOMP. We have demonstrated that a synthetic peptide vaccination against chlamydiosis is not an effective management tool in a koala population with a high prevalence of *C. pecorum* infection and related disease. The lack of antigenic response found in this study suggests that further research utilising a larger, full-length antigen is an avenue worth investigation if we are to consider vaccination as a part of a management strategy in diseased koala populations.

## Introduction

Koalas (*Phascolarctos cinereus*) have been listed as an endangered species in the northern parts of their range in the Australian states of New South Wales, Queensland and the Australian Capital Territory^1^, as these populations are forecast to decline by approximately 50% over three generations^2^. One of the major factors contributing to this decline is infection with *Chlamydia pecorum*^3^, with some reports of prevalence within free-ranging populations ranging from 58 to 66% within New South Wales^4,5^. Chlamydiosis, the resultant disease of *C. pecorum* infection, causes conjunctivitis, cystitis^6^ and reproductive tract pathology of females and males^7,8^ which can result in infertility, the main mechanism of population decline^9^. Infected females often exhibiting paraovarian cysts (up to 63% of cases)^10^ and males also showing sperm abnormalities^11^.

Options to address *C. pecorum* infection in wild koala populations are limited due to the duration and side effects of antibiotic therapy^12^. Strategic vaccination therefore offers potential as an alternative management tool, to reduce transmission and limit progression of disease. In murine models, vaccination has been shown to induce protection against the development of infertility in female mice infected with *Chlamydia muridarum*^13^. Previous studies investigating a *C. pecorum* vaccine in koalas have shown that vaccination can elicit humoral and cell-mediated immune responses^14–17^. Most studies examining vaccine induced immune responses have been in disease free koalas^14–20^. Vaccine efficacy has been assessed in wild koalas with low levels of *C. pecorum* prevelance^21,22^ and koalas with mild ocular disease^23–25^, however the effect of vaccination in moderate to severe or chronic disease has not been thoroughly investigated. Vaccination may be of limited direct benefit to individuals in chronically diseased populations, as chlamydiosis induces a chronic inflammatory response and structural disease comprising proliferation and fibrosis of the conjunctiva^6^, fibrosis of the salpingeal ducts, uteri and cervices^7^, and formation of paraovarian cysts^26,27^, most of which is expected to be irreversible^28^. In these populations, however, vaccination may have a role in reducing transmission by reducing chlamydial shedding in infected animals, but its potential benefits need to be further evaluated before widespread implementation.

To determine if vaccination is a valuable tool to address disease in wild koala populations, data on long-term efficacy also needs to be established. The majority of vaccine studies in koalas have monitored responses up to 6 months post vaccination^14,18,20,21,23–25,29^. To be a part of a feasible strategy to address chlamydiosis in wild koala populations, vaccination should provide beneficial effects for greater than 6 months. Specifically, vaccination should aim to reduce chlamydial shedding in infected individuals to reduce transmission events and reduce the development or progression of reproductive pathology. Koala chlamydial vaccine efficacy has been monitored for up to 12 months^22^ but despite immune responses persisting, beneficial effects including a reduction in chlamydial shedding or prevention of clinical disease progression has not been shown beyond 6 months post vaccination^21,22^.

Production of the antigen within the existing recombinant protein-based vaccine for widespread use is challenging. An alternative that addresses the difficulties of production is use of synthetic peptide antigens within the vaccine. Peptide based antigens are more cost effective, easier to produce, are stable for longer term storage and have been shown to stimulate immune responses and reduced chlamydial shedding in mice^30–32^. A synthetic peptide vaccination with two epitopes of the major outer membrane protein (MOMP) has been trialled in disease-free koalas. This vaccination showed a synthetic peptide vaccine could stimulated humoral and cell-mediated immune responses^18^.

To evaluate a synthetic peptide vaccine as a management tool in wild koala populations, this study investigates vaccine efficacy, measured by changes to disease expression, chlamydial shedding and humoral and cell mediated immune responses, over 18-months in a koala population with a high prevalence of chlamydiosis in a blinded randomised placebo-controlled vaccination trial.

## Materials and methods

### Ethics

All handling and sampling of koalas was performed by experienced veterinarians under the University of Sydney Animal Ethics Approval Number 2019/1547 and NPWS Scientific Licence SL102331. All methods were performed in accordance with the relevant guidelines and regulations.

### Cohort recruitment

Koalas recruited to this study were captured within a 15 km radius from − 31.159855, 150.118456, south–west of the town of Gunnedah, New South Wales, using the ‘noose and flag’ technique^33^. Alfaxalone (Jurox), was used to sedate the koalas at a dose rate of 1.8 mg/kg by intramuscular injection and then oxygen and isoflurane was administered to effect through a fitted mask to anaesthetise the koala. The same veterinarian (SJS) assessed and sampled all koalas. At initial assessment, koalas were classified as either diseased or non-diseased, based on external signs of chlamydiosis, (presence of a “wet bottom” or ocular disease)^34^. These criteria were chosen as these clinical signs can improve with veterinary treatment^35^ and infection status of individuals was unknown at initial capture. Following this classification, the koala was randomly assigned to one of two treatment groups, vaccination or placebo. The veterinarian who examined the koalas and administered the treatment was blinded to treatment group allocation. VHF collars (transmitter model M3420, ATS Australia) were fitted to the koalas so they could be radio-tracked and visually monitored from a distance at 2-month intervals throughout the study, and recaptured and clinically evaluated at 6, 12 and 18 months following vaccination.

### Koala clinical examination

The following procedures were performed at timepoint 0 (recruitment), 6, 12 and 18 months post vaccination. Digital images were taken of the koala’s identification tag, eyes, teeth (for age estimation)^36^ and perineal region. Koalas were weighed and a body condition score (BCS) ranging from 1 to 5 was recorded^37^. Both eyes were assessed for ocular discharge, conjunctival proliferation, and conjunctival chemosis, each criterion with a score between 0 and 3 allocated to each eye^34^. The perineal region was examined for urine leakage, erythema, or the presence of purulent discharge. A wet bottom score was allocated, based on a modification of the Griffith^34^ scoring system, whereby only scores 0–6 were used because scores 7–9 required prolonged observation, not amenable to field examinations. Ultrasonography was performed with the koala in dorsal recumbency^38–40^. For ultrasonographic examination of the kidneys, a small patch of fur was clipped on the abdomen ventral to the approximate site of each kidney. Both kidneys were identified and the presence of any structural abnormalities such as dilated renal pelvis, dilated ureters or presence of renal calculi were recorded. For females, the transducer was placed inside the pouch and female reproductive structures visualised by scanning the region cranio-lateral to the bladder. If paraovarian cysts were identified, it was noted if the cyst/s were unilateral, bilateral, multiloculated or singular. A paraovarian cyst score was allocated with 0 = no abnormalities, 1 = unilateral paraovarian cysts identified and 2 = bilateral paraovarian cysts identified. If paraovarian cysts were not detected, a thorough scan of the caudal abdomen was performed to confirm their absence. In males, ultrasound was performed to identify the prostate by directing the transducer caudo-ventral from the bladder. Ultrasound was also performed on both testes.

Swabs for detection of *C. pecorum* DNA were collected from the conjunctiva bilaterally and the female urogenital sinus or male urethra (Copan, Interpath, 160C). For conjunctival sampling, swabs were placed into the conjunctival fornix and rotated five times. In females, swabs were inserted 2–3 cm into the urogenital sinus and rotated five times. In males, the penis was everted, and the swab placed 2 cm into the urethra and rotated five times. All swabs were stored at − 20 °C until processing.

### Vaccination

Four peptides from *C. pecorum* MOMP, were used as the antigens within the vaccine, P1 H-EGMSGDPCDPCATW-OH, P2 H-INYHEWQVGAALSYRLNMLIP-OH, utilised previously^18^, P3 H-VLQIVSLQINKLKSRKACG-OH, P4 H-KKLLKSAFLSAAFFAG-OH, representative of the fifth conserved region and the leader sequence of MOMP, respectively. All peptides were synthetised by Mimotopes (Melbourne, Australia), at a purity of > 70% determined by high pressure liquid chromatography. Two of these peptides were chosen as they have been shown to stimulate humoral and cell mediated immune responses in healthy koalas following vaccination^18^. The additional two peptides were selected as they represent identified B-cell vaccine specific epitopes of conserved regions within *C. pecorum* MOMP that have been recognised by koalas, following MOMP antigen vaccination^25^. Kyte-Doolittle plots were constructed to determine hydrophobic and hydrophilic regions of each four peptides. The antigen was combined with a three-component adjuvant, containing Poly I:C (250 μg), Host Defence Peptide-Innate Defence Regulator IDR-1002 (500 μg), and Polyphosphazene EP3 (250 μg) (VIDO-Intervac, University of Saskatchewan, Canada). Vaccine preparation involved mixing the Poly I:C and IDR-1002 and incubating for 15 min at room temperature, with gentle rocking. Next, EP3 and the four peptides were added, with the final mixture incubated for a further 15 min at room temperature, with gentle rocking, then stored at 4 °C until use^25^. Vaccination and placebo injections (Hartmann’s solution) were administered subcutaneously (0.5 ml) into the interscapular region at first capture and then again at 6 months.

### Urogenital and ocular swab DNA extraction and qPCR

DNA was extracted from ocular and urogenital swabs using the MagMAX™ CORE Nucleic Acid Purification Kit #A32702. A sterile swab was included as a control to monitor for contamination during the extraction process. A multiplex qPCR assay was used to quantify the following genes from each extracted swab sample, *P. cinereus beta actin* (HEX), *23S Chlamydia* (ROX) and *C. pecorum ompB* (FAM); primers adapted from Hulse et al*.*^41^. PCR reactions were made to a final volume of 20 µl consisting of 10 µl of SensiFAST™ Probe, 400 nM of each primer, 200 nM of each probe, 4.4 µl of dH_2_O and 2 µl of DNA. The cycling conditions included initial denaturation for 2 min at 98 °C, followed by 40 cycles of denaturation for 15 s at 98 °C and a combined annealing and extension step for 30 s at 58 °C. Samples were determined to be *C. pecorum* positive if the CT value of *C. pecorum ompB* was ≤ 34. Samples were determined to be suspect *C. pecorum* positive if the CT value of *C. pecorum ompB* was 34–40 and the *23S Chlamydia* was 34–40. qPCR assay was used to quantify *P. cinereus beta actin* and *C. pecorum ompB* genes.

### Plasma anti-major outer membrane protein (MOMP) IgG ELISA

All concentrations of reagents, blocking and incubation conditions were determined by optimisation experiments using chequerboard titrations. In the optimised assay, 96 well flat bottom plates (Grenier #0030125150) were coated with 2 µg of recombinant MOMP serovar G (produced utilising methods previously described)^14^ in carbonate-bicarbonate buffer (Sigma-Aldrich #C3041) at 100 µl/well at 4 °C overnight. Wells were emptied and then blocked with 300 µl of 5% skim milk powder in PBS with Tween (0.05%) (PBST) at 37 °C for 1 h. Wells were emptied and koala plasma was applied to the wells at a concentration of 1:400 at 100 µl/well and incubated at 37 °C for 1 h. Wells were then emptied and washed five times with PBST with a microplate washer (Biorad model 1575). An in-house sheep anti-koala-IgG antibody^18^ 100 µl was added to each well at a dilution of 1:8000 and incubated at 37 °C for 1 h. Following incubation, wells were washed as described above. 100 µl of rabbit anti-sheep IgG HRP conjugated (Abcam #ab6747) was added to each well (1:20,000 dilution) and incubated at 37 °C for 1 h. Following incubation, wells were washed as described above, using PBS only. Finally, 100 µl of 3,3′,5,5′Tetramethylbenzidine (Sigma-Aldrich #T5525) was added to each well and allowed to develop at room temperature, in the dark, for 20 min before 100 µl of 1 M H_2_SO_4_ was added to each well. Optical density (OD) was read at 450 nm. Each plate contained a blank well (no antigen or plasma), the same negative control from a captive, *C. pecorum* negative koala and a standard curve based on doubling dilutions of a known strong positive sample, selected during optimisation, from a *C. pecorum* positive koala. The inter-assay coefficient of variation was < 15% and the intra-assay coefficient of variation was accepted if it was < 10%. Each sample was run in two wells coated with antigen and two without antigen (four wells total). All samples were run in duplicate individual wells, with one well coated with antigen and one without antigen, and the mean was calculated from these replicates. All OD values were blank adjusted; the OD value of the no-antigen well was subtracted from the OD value of the antigen and plasma well for each sample. The highest concentration standard was given a nominal value of 32. The other values were calculated relative to that standard, based on dilution. The OD values of the samples were compared to the standard curve using a 4-parameter logistic curve.

### Lymphocyte stimulations

Three millilitres RPMI medium (Sigma-Aldrich #R7388), was incubated for 30 min at 37 °C. The same volume of heparanised blood from each koala was centrifuged at 3000 g for 5 min and the leukocyte fraction (buffy coat) was aspirated and suspended in the pre-incubated media. In duplicate, 5 µg of rMOMP genotype G in 25 µl of foetal calf serum (FCS) or, in the case of negative controls FCS 25 µl, was added to 220 µl of the buffy coat solution. Suspensions were then incubated for 12 h at 37 °C then 750 µl of RNA later (Sigma-Aldrich #R0901) was added to each sample and the sample stored at room temperature for 24 h, then frozen at − 20 °C until processing.

### RNA extraction and cDNA synthesis

RNA was extracted from the leukocyte suspensions using the RiboPure—Blood kit (Invitrogen #AM1928) according to the manufacturer’s protocol. Extracts were treated with RNAse-free DNAse, (Thermofisher #EN0521) and cDNA synthesis was then performed using the RevertAid First Strand cDNA Synthesis Kit (K1622) according to the manufacturer’s protocol.

### IFNγ and IL-17A expression

A qPCR assay was used to estimate expression of the following genes: GAPDH, IFNγ and IL-17A^42,43^. Reactions were made to a final volume of 20 µl consisting of 10 µl of SsoAdvanced™ Universal SYBR Green Supermix (BioRad), 0.5 µM of each primer with 6 µl of H_2_O (GAPDH) and 0.3 µM of primer with 6.8 µl of H_2_O (IFNγ and IL-17A) and 2 µl of DNA. Cycling conditions for the different genes were applied as previous^42,43^. All samples were run in duplicate with negative controls at each step. IFNγ and IL-17A expression for each koala for each time point were normalised to GAPDH by the 2^−ΔΔCT^ method, where ΔΔCT = (CT of target − CT of GAPDH) − (CT of target—CT of GAPDH) and presented as a fold change in relation to the unstimulated sample.

### Statistical analysis

All data analyses were performed using the R Statistical Environment (Version 3.6.1)^44^. Data were initially assessed using the ‘ggplot’ and ‘ggdensity’ functions from the ‘ggplot2’^45^ and ‘ggpubr’^46^ packages respectively. Shapiro–Wilk tests were used to check for normality. Data that did not conform to normality were log transformed^47^.

Spearman’s Rank Correlations were used to test for relationships between immunological markers, chlamydial shedding, and disease variables. A variable was removed if a pair of variables had a correlation coefficient > 0.5 (or < –0.5)^48^. To normalise *C. pecorum* CT against swab yield (koala beta-actin DNA) from the same swab sample, ΔCT values were generated (ΔCT = CT of *C. pecorum* CT of beta-actin). Chlamydial shedding is expressed as the inverse of *C. pecorum* ΔCT values because, as chlamydial shedding decreases, *C. pecorum* ΔCT values increase.

The effect of treatment (vaccination or placebo), time since vaccination, their interaction and sex, was tested with different linear mixed effects models (LMM), constructed using the ‘lme4’ package^49^, with individual koala as the random factor. Dependant variables examined included clinical disease variables, specifically ocular disease scores, wet bottom score and paraovarian cyst score (females only), chlamydial shedding (ocular and urogenital ΔCT values), anti-MOMP plasma IgG values (log transformed) and cytokine expression (log transformed fold change).

## Results

### Cohort recruitment

Fifty-two koalas were recruited into the study (Table 1). Post vaccination data were measured for all koalas at 6 months, 41 koalas at 12 months and 28 koalas at 18 months. The decrease in sample size was due to koalas dying from natural causes. The average tooth wear class at the beginning of the study was pre-molar four being “flat” with molar one having some wear which signifies an estimated average age of 9 years and above^36^. *C. pecorum* infection prevalence of the koalas sampled at the beginning of the study was 79% (Table 1).

**Table 1:** Cohort characteristics of enrolled study group at recruitment. Numbers indicate number of koalas.

	Placebo		Vaccine		Total	Percentage
	Female	Male	Female	Male		
Enrolled	13	11	17	11	52	
*C. pecorum* qPCR positive ocular site	0	0	1	1	2	3
*C. pecorum* qPCR negative ocular site	13	11	16	10	50	97
*C. pecorum* qPCR positive urogenital site	11	9	14	7	41	79
*C. pecorum* qPCR negative urogenital site	2	2	3	4	11	21
Ocular clinical disease	7	6	6	5	24	46
Perineal urine staining	5	0	5	2	12	52
Paraovarian cysts	10	NA	12	NA	22	73

### Ultrasonography

Repeated ultrasonography showed that the presence of paraovarian cysts can change over time. Unilateral or bilateral paraovarian cysts of five female koalas were not detected subsequently. The maximum size of any paraovarian cystic structure that was not identified on subsequent ultrasound was 55 mm in diameter. Four additional female koalas showed fluctuations over 18 months, whereby paraovarian cysts would be detected, not detected, and then detected again, sometimes on opposing sides.

### Vaccination response

No correlations were sufficiently strong to warrant exclusion of variables from the model (Supplementary Table S1), in supplementary material.

### Clinical disease

There was no significant effect of vaccination treatment (F_1,53 =_ 0.19, *P* = 0.665), time (F_3,113 =_ 2.14, *P* = 0.09), sex (F_1,73 =_ 0.39, *P* = 0.529) or the interaction of time and treatment (F_3,113 =_ 0.42, *P* = 0.733) on ocular disease scores. There was also no significant effect of vaccination treatment (F_1,51 =_ 0.07, *P* = 0.783), time (F_3,115 =_ 1.13, *P* = 0.337) or their interaction (F_3,115=_ 0.99, *P* = 0.397) on wet bottom score disease scores. Sex had a significant effect on wet bottom score, with females having a higher wet bottom score compared to males (F1,56 = 7.71 *P* = 0.007). In females, there was no significant effect of vaccination treatment (F_1,28_ = 0.63, *P* = 0.432), time (F_3,64_ = 0.48, *P* = 0.696) or their interaction (F_3,64_ = 0.51, *P* = 0.674) on paraovarian cyst score.

### Chlamydial shedding

#### Ocular site

**Figure 1:**
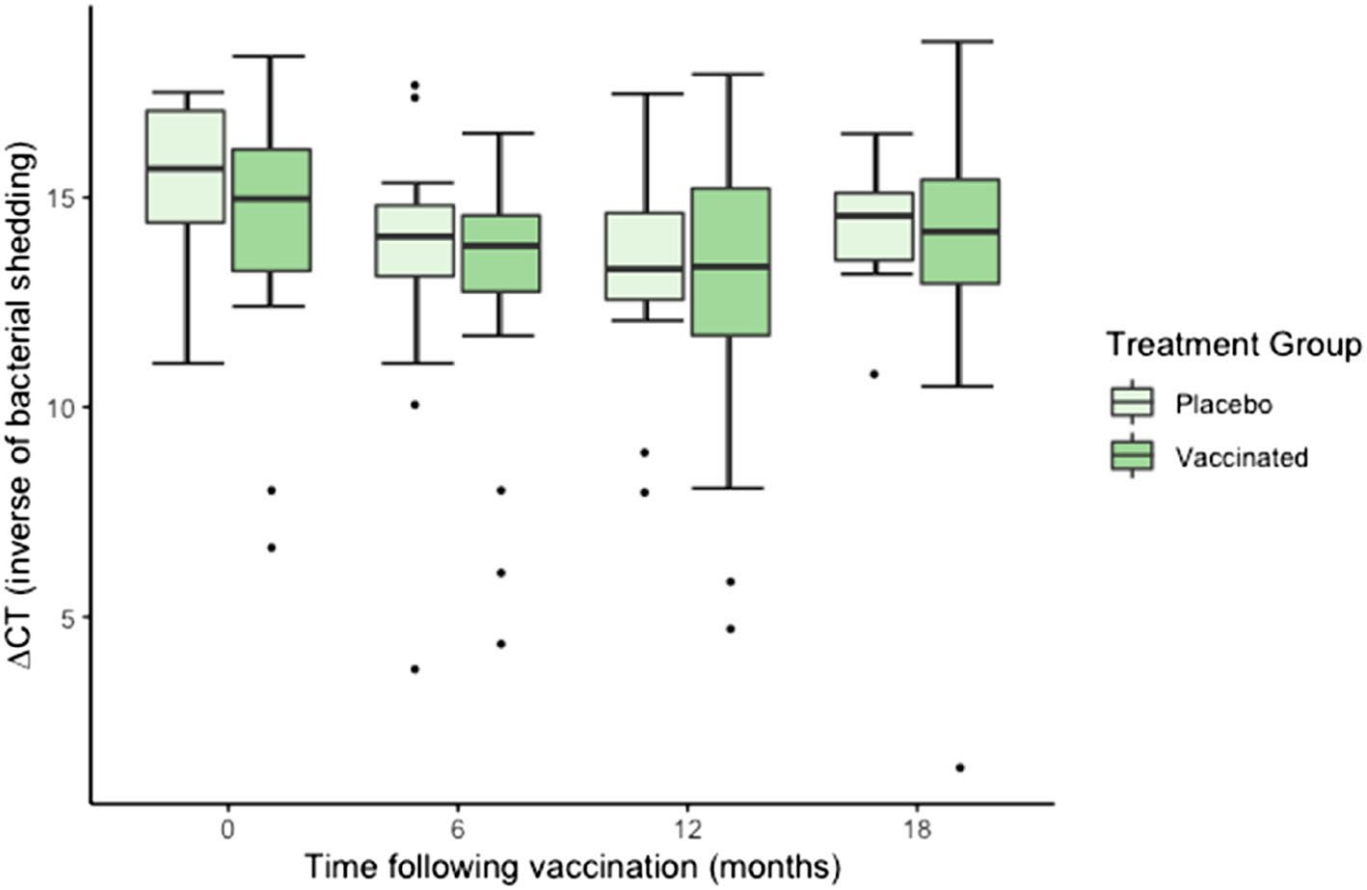
*C. pecorum* ∆CT values at the ocular site of vaccinated and placebo koalas, 0–18 months following vaccination (n = 52 at 0 and 6 months, n = 41 at 12 months and n = 28 at 18 months). Note: ∆CT reflects *C. pecorum* CT—β-Actin CT (normalising gene) whereby an increase in ∆CT reflects a decrease in chlamydial shedding.

There was no significant effect of vaccination treatment (F_1,55_ = 1.99, *P* = 0.163), sex (F_1,61_ = 0.71, *P* = 0.401) or the interaction of time and treatment (F_3,119_ = 0.59, *P* = 0.620) on *C. pecorum* ocular ∆CT values (Fig. 1). There was a significant effect of time (F_3,118_ = 9.49, *P* = 0.001): both vaccinated and placebo groups had higher ocular ∆CT values (i.e., less chlamydial shedding) at time zero.

#### Urogenital site

**Figure 2:**
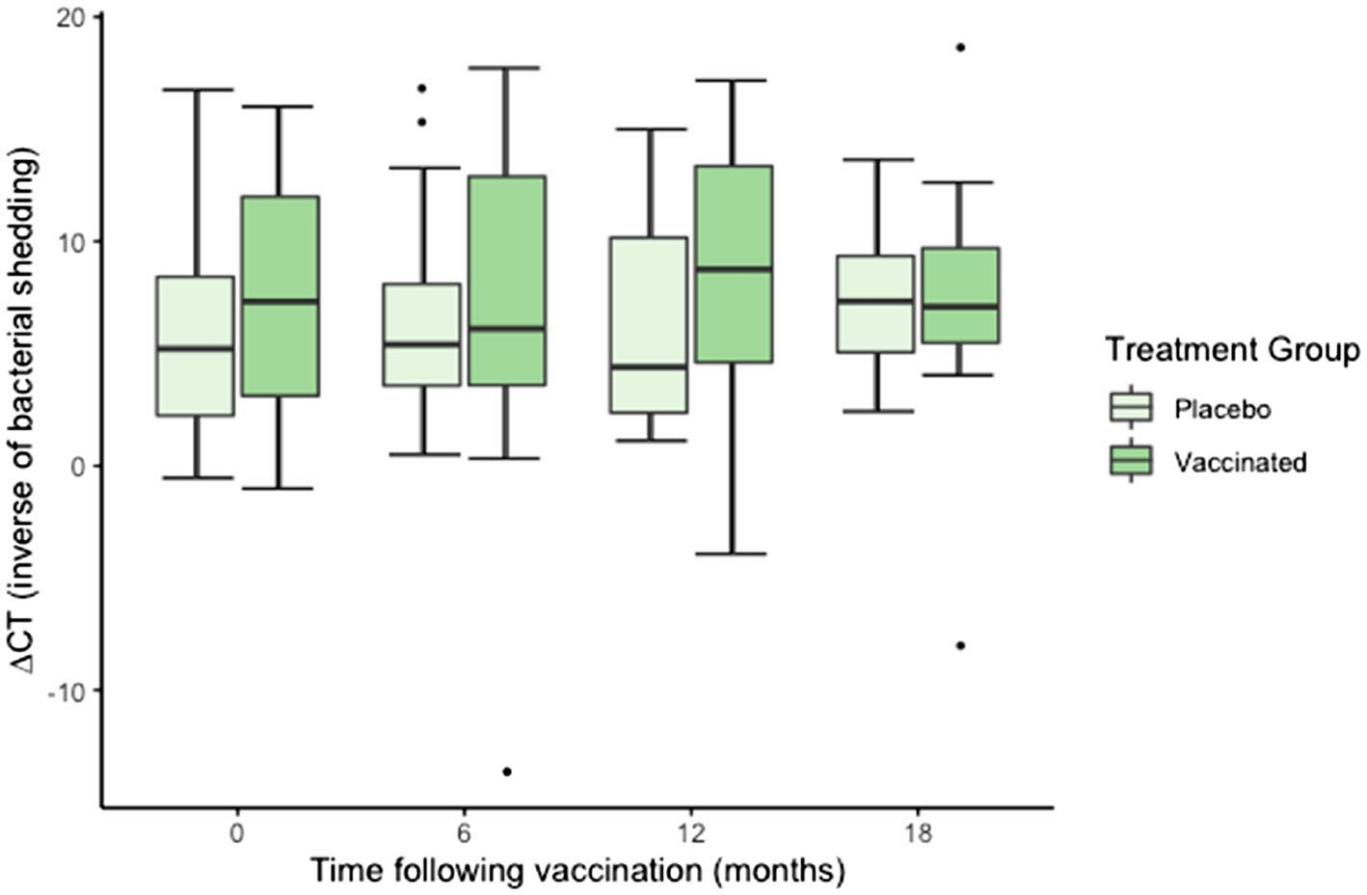
*C. pecorum* ∆CT values at the urogenital site of vaccinated and placebo koalas, 0–18 months following vaccination (n = 52 at 0 and 6 months, n = 41 at 12 months and n = 28 at 18 months). ∆CT reflects *C. pecorum* CT—β-Actin CT (normalising gene) whereby an increase in ∆CT reflects a decrease in chlamydial shedding.

There was no significant effect of vaccination treatment (F_1,55_ = 1.58, *P* = 0.213), time (F_3,119_ = 0.61, *P* = 0.603), sex (F_1,59_ = 1.54, *P* = 0.218) or the interaction of time and treatment (F_3,119_ = 0.36, *P* = 0.776) on *C. pecorum* urogenital ∆CT values (Fig. 2). Vaccination did not prevent the detection of new *C. pecorum* shedding at the urogenital site, as three vaccinated koalas that were negative on initial sampling, became positive over the 18 months. Four female koalas ceased shedding, however this seemed unrelated to vaccine status as two were vaccinated and two were not.

### Plasma anti-major outer membrane protein (MOMP) IgG ELISA

**Figure 3:**
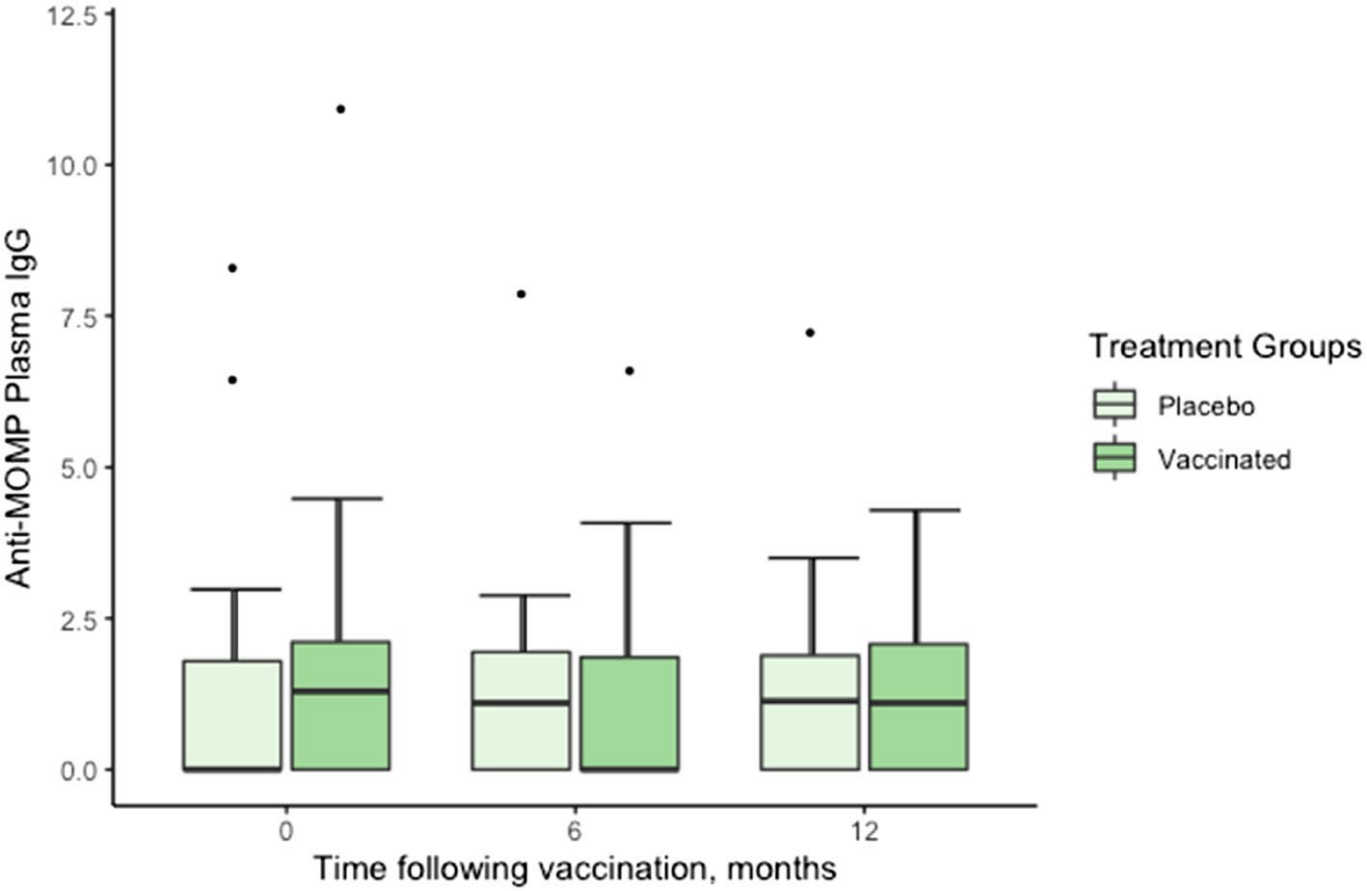
Plasma anti-MOMP IgG values of vaccinated and placebo koalas, 0–12 months following vaccination (n = 52 at 0 and 6 months, n = 41 at 12 months). Plasma anti-MOMP IgG values on the Y axis represent values derived from a 4-parameter logistic curve.

There was no significant effect of vaccination treatment (F_1,39_ = 0.05, *P* = 0.820), time (F_2,79_ = 0.09, *P* = 0.906), or the interaction of time and treatment (F_2,79_ = 1.25, *P* = 0.291) on anti-MOMP IgG levels (Fig. 3). Sex had a significant effect on anti-MOMP IgG values (F_1,39_ = 3.86, *P* = 0.050) with females having lower anti-MOMP IgG values compared to males.

### IFNγ and IL-17A mrNA expression from rMOMP stimulated peripheral blood lymphocytes

#### IFNγ

**Figure 4:**
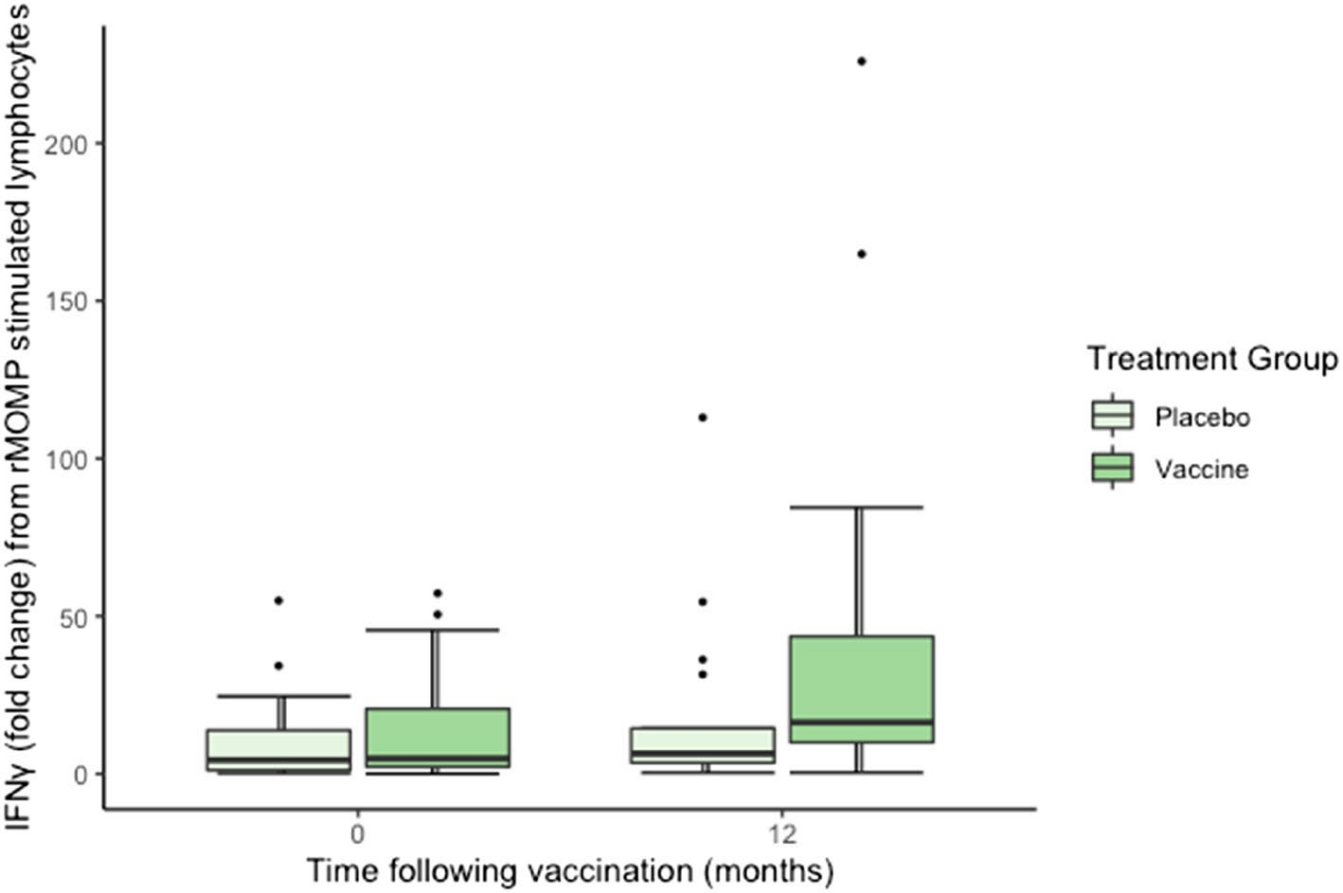
Fold change in IFNγ mRNA in response to rMOMP stimulation of peripheral blood lymphocytes of vaccinated and placebo koalas, 0–12 months following vaccination (n = 52 at 0 months and n = 41 at 12 months).

There was no significant effect of vaccination treatment (F_1,73_ = 2.90, *P* = 0.09), sex (F_1,73_ = 2.06, *P* = 0.155, or the interaction of time and treatment (F_1,73_ = 1.35, *P* = 0.247) on IFNγ mRNA expression from peripheral blood lymphocytes (Fig. 4). Time had a significant effect on IFNγ expression (F_1,73_ = 6,44, *P* = 0.013) where expression was higher 12 months post vaccination.

#### IL-17A

**Figure 5:**
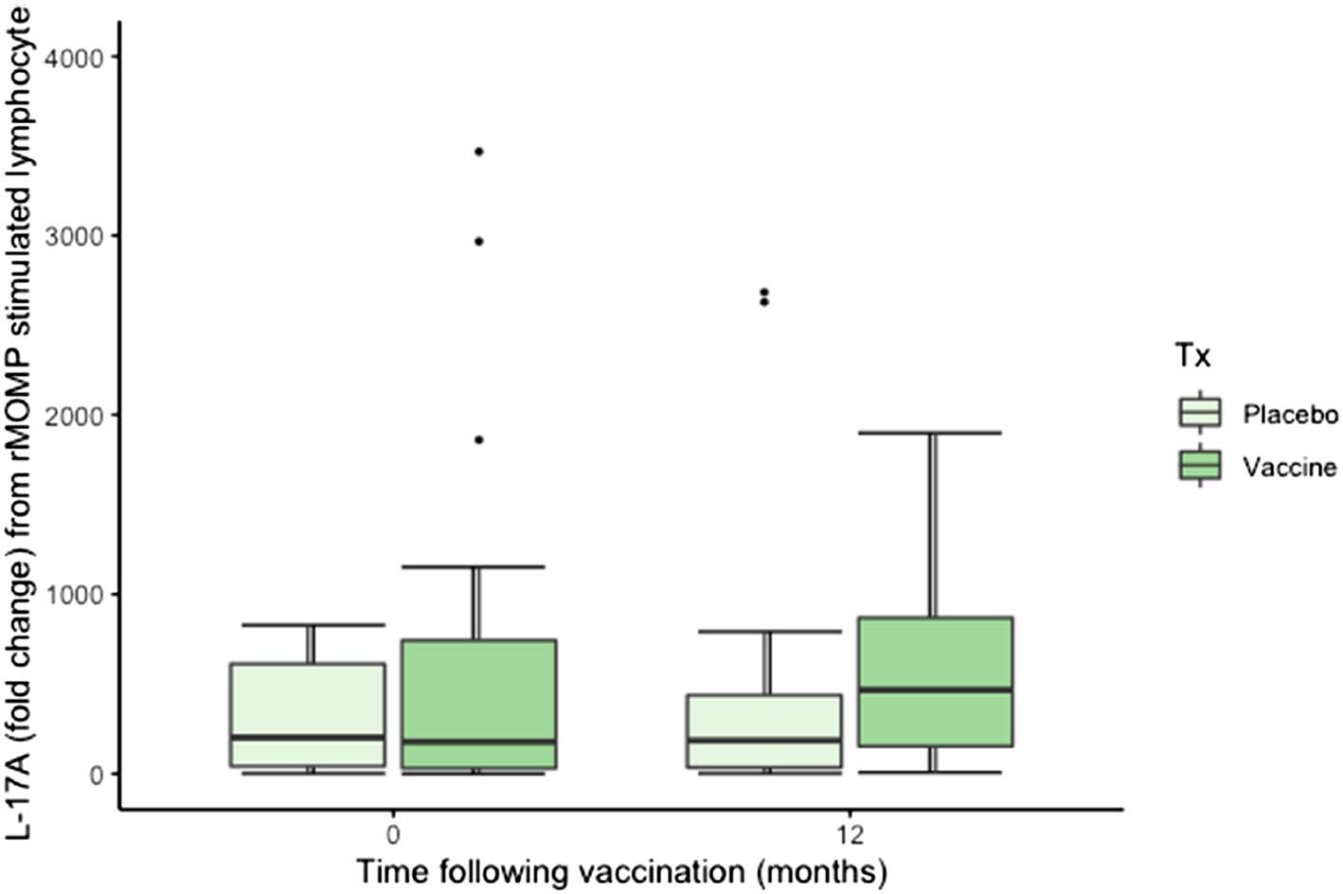
Fold change in IL-17A mRNA in response to rMOMP stimulation of MOMP stimulated peripheral blood lymphocytes of vaccinated and placebo koalas, 0–12 months following vaccination (n = 52 at 0 months and n = 41 at 12 months).

There was no significant effect of vaccination treatment (F_1,37_ = 0.09, *P* = 0.762), time (F_1,38_ = 0.4, *P* = 0.530), sex (F_1,35_ = 1.91, *P* = 0.175, or the interaction of time and treatment (F_1,38_ = 2.09, *P* = 0.155) on IL-17A mRNA expression from peripheral blood lymphocytes (Fig. 5).

## Discussion

Immunological markers, along with the degree of chlamydial shedding and clinical response parameters in this blinded randomised placebo-controlled vaccination trial, indicate that this synthetic peptide vaccination regime is not an effective management tool to manage chlamydiosis within a high disease prevalence koala population. At 6, 12 and 18 months post vaccination, there was no significant difference in chlamydial shedding, disease expression, plasma anti-MOMP IgG levels or antigen stimulated IFNγ or IL-17A expression between vaccinated and placebo koalas. Additional findings include apparent vaccine-independent resolution of paraovarian cysts and the low prevalence of chlamydial shedding among koalas with clinical ocular disease.

This synthetic peptide vaccine provided no identifiable reduction in chlamydial shedding or disease as measured by ocular, urogenital and paraovarian cyst scoring. The inability to demonstrate a reduction in chlamydial shedding or disease is likely a result of no identifiable vaccine-induced cell-mediated or humoral immune stimulation. The lack of response found in this study contrasts studies utilizing full length MOMP as a vaccine antigen in mice^50^, humans^51^ and koalas^21,22^. The antigen used in this study comprised of four synthetic peptides of the MOMP protein that ranged from 16 to 21 amino acids in length. There is evidence that synthetic peptide vaccines utilising MOMP peptide components can stimulate immune responses and reduced chlamydial shedding in mice^31,32,52^. Our contrasting results may be the result of the size of peptides; the peptides examined in murine studies were larger and thus likely to be a more immunogenic antigen^31^. An alternate possibility is that the antigens chosen did not represent immunogenic epitopes or that the peptide was not conformationally consistent with that of the full MOMP protein. We hypothesise that either of these two scenarios would result in a lack of immune recognition of the *C. pecorum* elementary body (EB) when the koala is infected. It is possible that a response occurred to the vaccine peptides but was not detected in our assays as the full length recombinant MOMP was used as an antigen in the ELISA and lymphocyte stimulations as it is this response that we hypothesised is needed for vaccine efficacy. Another alternative is that there was a transient response prior to first sampling at 6 months; previous synthetic peptide vaccine trials examined vaccine response over weeks rather than months^31^. Tifrea et al*.*^31^ detected an elevation in plasma anti-MOMP IgG and an increase in plasma IFN-γ at 4 weeks following vaccination, however, did not measure immune responses beyond this time. Therefore, it remains possible that the synthetic peptide vaccination provided a positive effect over the short term (weeks) but, as our study aimed to evaluate longer-term responses to build evidence on the use of widespread vaccination programs, short term vaccine response was out of scope of this study. While the vaccine protocol within this study included a single booster, it is plausible that the observed lack of response could stem from the need for additional booster vaccines. In studies involving other marsupials, multiple booster vaccinations have shown a tenfold rise in antibody levels^53^. However, Nyari et al*.*^18^ demonstrated that a single dose of a vaccine comprised of two synthetic peptides of the MOMP antigen stimulated a plasma anti-MOMP IgG response in healthy koalas. Therefore, it is likely that the characteristics of our population impacted our ability to demonstrate a vaccine induced immune response.

The demographics of this population may have contributed to the lack of development of vaccine induced immune responses. There is evidence of reduced fecundity within our study population, with only one breeding female observed within this cohort across two breeding seasons. This decline in fertility has coincided with an increase in *C. pecorum* prevalence within the population^5^. As a consequence of the reduced fecundity over a period of years, the population is geriatric, with the average estimated age from tooth class wear being 9 years and above^36^. Immunosenescence is well documented in humans and refers to an age-related dysfunction of immune cells^54^. One example of immunosenescence is the reduced antibody production and cell mediated responses (IFNγ) in geriatric patients following influenza vaccination^54^. In koalas, increased age, specifically 9 years and older, has been associated with a decrease in CD4 and CD8β responses^55^. The lack of immune response development in this study may be in part, therefore, a result of our study population demographics.

Koala populations with a high degree of pathology attributable to chronic inflammation may not be a suitable population to assess vaccine responses and vaccination may not be a suitable tool to address individual animal health effects however may provide a suitable tool to reduce chlamydial shedding and therefore reduce transmission events. Urogenital fibrotic inflammation that results from *C. pecorum* infection is likely somewhat irreversible^7^. In humans, severe persistent conjunctival inflammation following *C. trachomatis* infection, is associated with scarring and fibrosis^56^ and can persist despite medical intervention^57^. Histopathological assessment of koalas with severe ocular disease demonstrates conjunctival fibrosis^6^, which is unlikely to resolve with medical treatment. At the beginning of this study, 5% of the males and females showed evidence of severe conjunctival proliferation and 73% of the females had evidence of genital tract pathology (paraovarian cysts) attributable in some part to chronic inflammatory process such as fibrosis of the salpingeal ducts. This chronic pathology was previously thought to be a permanent structural change and therefore unlikely to be altered by medical treatment alone, including vaccination^7^. In populations severely impacted by chlamydiosis, vaccination may therefore be ineffective at altering disease outcomes. The results of this study suggest that reducing transmission events by sterilisation or culling of infected individuals may offer better potential as management tools in these severely affected populations^58^.

The sonographic resolution or change in paraovarian cysts in nine koalas, independent of vaccination, and monitored by a single operator, further supports the notion that, in at least some koalas, cyst formation is less likely to be primarily due to fibrotic occlusions and may have a more dynamic pathogenesis than previously thought^27^. Historically, due to strong associations between the two, studies investigating the pathogenesis of paraovarian cysts in koalas have hypothesised that paraovarian cysts develop due to fibrosis and occlusion of the salpingeal lumen and were presumed to be irreversible^7,59^. More recently, Pagliarani et al.^27^ demonstrated paraovarian cyst development in the absence of fibrotic inflammatory occlusions and hypothesised disruptions in epithelial homeostasis of the ovarian bursa due to *C. pecorum* infection was the main factor resulting in the accumulation of fluid in cystic ovarian bursae. Our sonographic findings further support the evidence that paraovarian cysts have a dynamic pathogenesis that may not be solely associated with fibrosis of the reproductive tract, and yet were still not influenced by the vaccine treatment in this study.

Within this population there was a disparity between clinical ocular disease and infection status, measured by qPCR. At the commencement of this study 3% of this population tested positive for *C. pecorum* at the ocular site despite 46% demonstrating clinical ocular disease. Within this population, higher disease scores correlated to severe proliferation of the conjunctiva, which likely represents the chronic stages of inflammation. The low sensitivity is likely a result of the fact that chronic inflammation and clinical signs can persist despite clearance of infection. This finding is consistent with humans with clinical trachomatis^60^ and the demonstration of persistent clinical signs despite clearance of infection following antibiotic therapy^61^. Another possibility for this low sensitivity is the incorrect classification of signs such as mild ocular discharge classified to be solely as a result of *C. pecorum* infection, rather than other pathogenesis.

We have demonstrated that a synthetic peptide vaccination against chlamydiosis is not an effective management tool in an aging koala population with a high prevalence of *C. pecorum* infection and related disease. Vaccination failed to achieve a reduction in chlamydial shedding and therefore is unlikely to reduce transmission of infection in this setting. The lack of antigenic response found in this study suggests that further research utilising a larger, full-length antigen warrants further investigation if we are to consider vaccination as a part of a management strategy in diseased populations. Although the demographics and chronic pathology exhibited by our population may have impacted the assessment of vaccine efficacy, the scenario is, nonetheless, typical of many koala populations for which vaccination might be considered desirable as a management option. Evaluation of future full length antigen vaccines should include investigation of longer-term efficacy, surpassing 6 months of vaccination, in wild koala populations less severely affected by chlamydiosis. Once a viable vaccine is developed, disease and demographic status of the population should be considered, and context-specific efficacy evaluated, prior to incorporating vaccine into population wide disease management plans.

### Supplementary Information

Supplementary Table S1.

## Data Availability

The data that support the findings of this study is available from the corresponding author upon request. There are no restrictions on data availability. This study is reported in accordance with ARRIVE guidelines.
