# Supplementary material for: Efficacy of a synthetic peptide Chlamydia pecorum major outer membrane protein vaccine in a wild koala (Phascolarctos cinereus) population
Source: Sci Rep. 2023 Sep 12;13:15087. doi: 10.1038/s41598-023-42296-7 (PMC10497537; doi:10.1038/s41598-023-42296-7)
Supplement: Supplementary file 1 — Supplementary Table S1. [file 41598_2023_42296_MOESM1_ESM.docx]

|  | Body Mass | BCS | Total eye score | Wet bottom score | Cyst grade | Bladder wall thickness | ΔCT Eyes | ΔCT urogenital | IFN*γ* | IL-17A | IgG |
| --- | --- | --- | --- | --- | --- | --- | --- | --- | --- | --- | --- |
| Body Mass | r = 1 |  |  |  |  |  |  |  |  |  |  |
| BCS | r = 0.05  p = 0.48 |  |  |  |  |  |  |  |  |  |  |
| Total eye score | **r = 0.18**  **p = 0.02** | **r = -0.19 p = 0.01** |  |  |  |  |  |  |  |  |  |
| Wet bottom score | **r = -0.23 p = 0.001** | **r = -0.17 p = 0.02** | r = 0.12  p = 0.11 |  |  |  |  |  |  |  |  |
| Cyst grade | r = 0.09  p = 0.43 | r = 0.01  p = 0.98 | **r = 0.20**  **p = 0.04** | **r = 0.24**  **p = 0.01** |  |  |  |  |  |  |  |
| Bladder wall thickness | r = 0.01  p= 0.87 | r = -0.01  p = 0.92 | r = 0.03  p = 0.71 | r = 0.00  p = 0.93 | r = 0.00  p = 0.06 |  |  |  |  |  |  |
| ΔCT Eyes | r = 0.01  p = 0.90 | r = 0.06  p = 0.45 | **r = -0.15 p = 0.05** | r = 0.01  p = 0.81 | r = 0.12  p = 0.23 | r = -0.10  p = 0.28 |  |  |  |  |  |
| ΔCT urogenital | r = -0.01  p =0.90 | r = 0.03  p = 0.67 | r = -0.17 p = 0.31 | **r = -0.16 p = 0.03** | r = -0.12  p = 0.40 | r = -0.09  p = 0.27 | r = 0.00  p = 0.26 |  |  |  |  |
| IFN*γ* | r = -0.11  p = 0.32 | r = -0.14  p = 0.23 | r = -0.10  p = 0.40 | r = -0.14  p = 0.43 | r = -0.09  p = 0.13 | r = -0.02  p = 0.83 | **r = -0.22 p = 0.001** | r = 0.14  p = 0.22 |  |  |  |
| IL-17A | r = 0.11  p = 0.31 | r = -0.16  p = 0.17 | r = -0.16  p = 0.88 | r = -0.13  p = 0.24 | r = -0.12  p = 0.64 | r = -0.13  p = 0.28 | r = 0.24  p = 0.28 | r = 0.15  p = 0.18 | r = 0.19  p = 0.10 |  |  |
| IgG | **r = 0.28**  **p = 0.00** | r = 0.16  p = 0.06 | r = -0.24 p = 0.06 | **r = -0.27 p = 0.001** | r = -0.02  p = 0.16 | r = 0.12  p = 0.15 | r = 0.22 p = 0.80 | r = 0.03  p = 0.73 | r = 0.02  p = 0.83 | r = 0.03  p = 0.84 | r = 1 |

**Supplementary Table S1: Spearman’s Rank Correlations Matrix between immunological markers, chlamydial shedding (ΔCT values) and disease variables. Correlations that are statistically significant are in bold; all represent weak linear relationships. Note: an increase in ∆CT reflects a decrease in chlamydial shedding.**
